# Children’s Physical Activity During the COVID-19 Lockdown: A Cross Cultural Comparison Between Portugal, Brazil and Italy

**DOI:** 10.1177/00315125231152662

**Published:** 2023-02-06

**Authors:** André Pombo, Carlos Luz, Luis P. Rodrigues, Cristina dos Santos C. de Sá, Cristhina Bonilha Huster Siegle, Patrizia Tortella, Guido Fumagalli, Rita Cordovil

**Affiliations:** 1 Escola Superior de Educação, Instituto Politécnico de Lisboa, Lisboa, Portugal; 2 Research Center in Sports Performance, Recreation, Innovation and Technology, SPRINT, Melgaço, Portugal; 3 Centro Interdisciplinar de Estudos Educacionais, Lisboa, Portugal; 4 Escola Superior Desporto e Lazer de Melgaço, Instituto Politécnico de Viana do Castelo, Melgaço, Portugal; 5 Research Center in Sports Sciences Health Sciences and Human Development, CIDESD, Vila Real, Portugal; 6 Departamento de Ciências do Movimento Humano, 58804 Universidade Federal de São Paulo, Sao Paulo, Brasil; 7 Faculty of Education, 18956 Free University of Bolzano, Bolzano, Italy; 8 Center for Research in Child Motor Development, 19051 University of Verona, Verona, Italy; 9 CIPER, Faculdade de Motricidade Humana, 37809 Universidade de Lisboa, Lisboa, Portugal

**Keywords:** lockdown, screen time, sedentary time, physical activity, motor development

## Abstract

The COVID-19 pandemic forced governments to implement measures that disrupted the daily routines of many families worldwide. We studied how the COVID-19 lockdown affected children’s routines in Portugal (PT), Brazil (BR), and Italy (IT) to determine if children’s age and country impacted their physical activity (PA) and sedentary time. We launched an anonymous online survey to assess how 3–12 years old children adjusted their daily routines to this situation. Parents reported the times each child was engaged in different activities throughout the day, and we used these data to calculate separately overall sedentary and physical activity time. We conducted separate analyses of variance for age and country on the percentage of time spent in the different activities. Results, based on the data from 3045 children in these three countries (PT *n* = 2044; BR *n* = 836; IT *n* = 165), showed that, during lockdown, most children spent most of their awake daily hours in sedentary activities. There was a clear age effect on the way their routines were organized. Percentages of time spent in intellectual activity, playful screen activity, and overall sedentary time were greater in the older age groups, whereas percentages of time spent in play (with and without PA) and in overall PA were greater in the younger groups. We found a main effect of country for all variables except play without PA. The country effect was mainly due to the difference between the routines in BR when compared to PT and IT. Values of playful screen time and overall sedentary activity were higher in BR than in the two European countries. Conversely, values for play with PA, PA, and overall PA (except in the older group) were lower in BR. Patterns of time spent in these activities were similar in IT and PT, but PA and overall PA times were higher in the two younger age groups in IT. In summary, percentage of PA time of confined children was low and decreased with age across all three countries and was particularly low for children in BR relative to those in PT and IT.

## Introduction

Since being declared a public health emergency of international concern (Huang et al., 2020), the COVID-19 pandemic has rattled daily life routines of many families worldwide. As a highly contagious virus (Harrington et al., 2016) without effective treatments, COVID-19 precipitated social isolation and lockdown measures as the best way to control the infection spread (Sun et al., 2020). Although many countries announced population lockdown measures, different governments varied in their restrictions. Hubei province in China, was placed under lockdown approximately three weeks after the start of the COVID-19 outbreak, resulting in travel restrictions, social distancing from cancelled events and gatherings, and closings of public places as well as schools and universities. Additionally, outdoor activities were limited to a maximum of 30 minutes every second day (Lau et al., 2020).

In Italy, the first western country seriously hit by the pandemic, prevention measures were taken in late January. Both health-checkpoints in airports and a flight ban from China rapidly escalated when the first autochthonous cases were detected in the Lombardy Region (February 21, 2020). Italian authorities first created a “red-zone” in a limited area (Northern Italy) and suspended all public events or sites open to the public, including schools (all levels), public places, gyms, and other places of aggregation (Decree-Law *n*. 6: February 23, 2020). This situation, rapidly changed into a severe mitigation response, including a lockdown of the entire country, from March 9 to May 3, 2020. Numerous decrees were promulgated, and, on March 11, 2020, the “I’m staying home” decree was issued. Commercial, educational, and sports activities were suspended. Measures become more and more restrictive, and people could only leave home for serious and documented reasons.

Portugal responded quickly to the coming menace. The first case was reported in the last week of February (2020) and, on March 16th, schools, companies, and non-essential public services across the country were closed. Two days later, a state of emergency was declared. School programs were transferred to a mixed system of broadcast television and on-line teaching from April to June, and all sports/leisure activities were suspended until September. Although quick walks and outdoor play time (20 min) were permitted during the confinement, all outdoor playground facilities were closed; and children were encouraged not to engage in any kind of contact with others. In this situation, without any organized physical activity (PA), free playtime outdoor, or opportunities to play with friends, Portuguese children diminished their PA behaviors, increased their screen time (Pombo, Luz, Rodrigues et al., 2021), worsened their eating habits (Direção-Geral da Saúde, 2020) and even decreased their motor competence levels (Pombo, Luz, de Sá et al., 2021).

On March 27, 2020, Brazil announced a temporary ban on foreign air travelers and most state governors imposed isolation policies. However, these isolation policies differed across different regions of the country, due to their social and cultural differences. Despite the differences, schools were closed across the country (Aquino et al., 2020). Access to parks, beaches, clubs, gyms, and other places of aggregation were also banned in several regions, interrupting the practice of PA/leisure by children and their families. This situation reduced PA practices of Brazilian children and increased their screen time (Sá et al., 2020). Children in confinement presented more sedentary and playful screen time (not school related screen time), and lower PA time (Harrington et al., 2016; Mittal et al., 2020; Moore et al., 2020; Pietrobelli et al., 2020; Pombo, Luz, Rodrigues et al., 2021; Sekulic et al., 2020; Sá et al., 2020). Varied living conditions were associated with varied effects on children’s PA in that having an outdoor space at home, living with other children, and belonging to a family with at least one adult free of working from home was associated with a child’s greater activity (Pombo et al., 2020). Italy and Portugal responded similarly to the spread of the disease, with both imposing nationwide lockdowns and closing schools, universities and all non-essential businesses. As noted, Brazil imposed different measures in different states; while physical distancing and confinement measures were implemented by the Brazilian government, no nationwide lockdown was imposed. Notwithstanding, Brazilian schools were closed, giving children very similar conditions to those of their European peers. This situation caused low amounts PA and high amounts sedentary time in children’s lives, with a potentially negative impact on children’s motor competence (Vandorpe et al., 2011), body composition, and cardiovascular fitness (Tomkinson & Olds, 2007).

Apart from the influences of the COVID-19 pandemic, western societies have been significantly less physically active over the past four decades than were past generations (Chau et al., 2017; Hallal et al., 2012; Huotari et al., 2010; Myers et al., 2015; Pucci et al., 2017). Currently, 45% of the European population never practice exercise and only 6% do so regularly (Eurobarometer 525, 2022); and, although 79.4% of European children play actively for at least one hour per day, only 50% use active transport (walking or cycling) to get to and from school (World Health Organization, 2020). The same trend can be found on the American continent where less than one-quarter (24%) of children 6–17 years of age participate in 60 minutes of PA daily (Merlo et al., 2020).

PA can assist in the development of high bone density, high levels of motor competence, better physical fitness, and healthy weight. Also, PA promotes children’s mental health, psychosocial skills, academic performance (Biddle et al., 2004) and a more robust immune system (Lasselin et al., 2016), considered essential to facing the pandemic situation. Removing movement opportunities from children’s daily life certainly diminishes PA health benefits, but cultural influences on this effect are uncertain. Our aim in this study was to explore possible cross-cultural differences caused by the COVID-19 lockdown on PA and sedentary time among children of Portugal, Brazil and Italy. We hypothesized that, during this period, all children would spend more time in sedentary, intellectual, and screen time behaviors than in PA, play, and outdoor time, independently of their country. We sought to explore uncertain country to country differences, due to the varied cultural environments.

## Method

### Participants

The initial data for this survey research included 3045 parental responses regarding their younger at-home children (<13 years of age) during the second and beginning third weeks of confinement (between March 23 and April 1, 2020). The parental inclusion criteria were to have at least one child under 13 years of age and to be spending the confinement period at home. For informed consent, all participants read the information about the study and agreed with the study’s conditions by clicking to proceed on the first page of the survey. Participants could withdraw at any time by not proceeding or not submitting the online survey. The survey and research project were approved by the Faculty of Human Kinetics ethics committee for Portugal and Italy (CEIFMH n^o^.6/2020) and by the Federal University of Sao Paulo for Brazil (CEP/UNIFESP n^o^.0413/2020).

After cleaning the database for any second time responses and for missing or obviously wrong information (e.g., more than 24 hours reported in a day, or no sleep time reported for children), data regarding 3045 children under age 13 (2044 Portuguese, 165 Italian and 836 Brazilian), subdivided into three age groups (3–5 years–1251, PT = 885, IT = 71, BR = 295; 6–9 years–1195, PT = 737, IT = 92, BR = 366; and 10–12 years–599, PT = 399, IT = 25; BR = 175) were used in this study. Descriptive statistics of the families who participated in the study are presented in Table 1.

**Table 1.** Frequencies of the Participating Families Across the Three Countries Studied.

		Portugal, %	Italy, %	Brazil, %
Number of people in my household (include all ages)	2	4.8	2.1	8.8
	3 to 4	74.7	71.2	75
	More than 4	20.5	26.7	16.2
How many people in the household are children aged 12 years or younger?	1 to 2	88.7	77.5	92
	3 to 4	11	22.6	7.9
	More than 4	0.3	0	0.1
Considering the adults that are at staying home, how many are working from home?	All	31.9	26.3	37.5
	Some	35.6	27.1	45.1
	None	32.5	46.6	17.4
The housing you are in is	Apartment	59.5	0	54.2
	House	40.5	100	45.8
Number of rooms of the household	Studio	0.1	0	0.3
	1 to 2	26.9	3.4	33
	3 to 4	65.7	50	64.4
	More than 4	7.3	46.5	2.2

### Survey

To assess how children under 13 years of age were dealing with the COVID19 confinement situation, we created a survey on *LimeSurvey,* hosted on the *Faculty* of *Human Kinetics, University of Lisbon.* After a first validation of the questions by a group of five child development experts and a first pilot testing with 23 families, the survey was publicized through social media (Facebook, Instagram, WhatsApp), and by email, and it was launched online on March 23, 2020. The survey—completed by the parent/adult responsible for the child (ren)—took approximately five minutes to complete and included the following four sections:

1. Household (three items - numerical input, and single choice): These questions pertained to the composition of the household, the number of children and adults who were at home, and how many adults were working from home.
2. Housing characteristics (four items - single choice): These questions pertained to the type and characteristics of the house (e.g., apartment or detached house; number of rooms), the presence or absence of indoor space (gym or exercise room) or outdoor space (no outdoor space, small outdoor space - up to 12 m^2^; large outdoor space – more than 12 m^2^) for PA.
3. Household routines (six items - Likert scale questions): These questions pertained to the level of concern regarding the COVID-19 situation and the way routines were being adjusted (i.e., comparisons between time spent in different activities before and after confinement).
4. Children’s routines (16 items - single choice, and numerical input): These questions related to the characteristics of the target child (age, sex, PA before confinement, health status) and the time (reported in minutes) spent in different activities during the previous day.

For more information please check: https://inqueritos.fmh.ulisboa.pt/index.php/894232?lang=en.

### Variables

We analyzed five categories of activities:

(a) Intellectual activity (school assignments and online classes);
(b) Playful screen time (games, movies, social networks, internet, audio and video calls);
(c) Play without PA (reading, drawing, painting, board games, cards, Legos, etc.);
(d) Play with PA (hide and seek, jumping, tag, etc.);
(e) PA (organized PA indoors, PA outdoors, walking the dog).

The first three categories (Intellectual activity, playful screen time and play without PA) were aggregated to calculate overall sedentary time, and the last two categories (play with PA and PA) were used to calculate overall PA time. The PA values were then converted into a percentage of the total time reported for all categories, henceforth called Percentage of PA (%PA).

### Statistical Analysis

Data analyses were based on parents’ answers for 3045 children divided into three age groups according to Portuguese school levels corresponding to different stages of children’s development (early childhood, group 1 = 3–5 years, *n* = 1251; childhood, group 2 = 6–9 years, *n* = 1195 and pre-adolescence, group 3 = 10–12 years, *n* = 599). Due to non-homogeneity of variances, we used unequal number Tukey Honestly Significant difference (HSD) *post hoc* tests to further investigate significant interaction and main effects, with statistical significance set at *p <* .05. We conducted a priori power analysis using G∗Power3 (Faul et al., 2007), assuming two-tailed analyses of variance (ANOVA) to test the difference between three independent group means, a medium effect size (d = 0.50), and an alpha of 0.05. This calculation suggested a required sample size of 42 participants to achieve statistical power of 0.95.

We used descriptive statistics and frequency analysis to describe the children’s living environments and routines during this period. We analyzed five categories of activities: Intellectual activity (school assignments and online classes); Playful screen time (games, movies, social networks, internet, audio, and video calls); Play without physical activity (reading, drawing, painting, board games, cards, Legos, etc.); Play with physical activity (hide and seek, jumping, running, etc.); Physical activity (organized physical activity indoors, physical activity outdoors, walking the dog). The first three categories (intellectual activity, playful screen time, and play without physical activity) were designed to reflect overall sedentary time, and the last two categories (play with physical activity and physical activity) were to represent overall physical activity time.

Separate 3 × 3 ANOVAs (age group by countries) were performed to investigate how the different activities and routines of the confined children were being organized according to children’s age and country of residence. Since, during lockdown, all children were in the same constrained situation that did not encourage the practice of vigorous PA, we did not find the usual lower involvement in PA and higher engagement in sedentary behaviors by girls (Fu et al., 2016; Kann et al., 2018; Telford et al., 2016), but found equivalent behaviors across sex as had several others (Bonilha et al., 2021; Pombo, Luz, de Sá et al., 2021). For that reason, sex was not included in this analysis.

## Results

**Figure 1.**
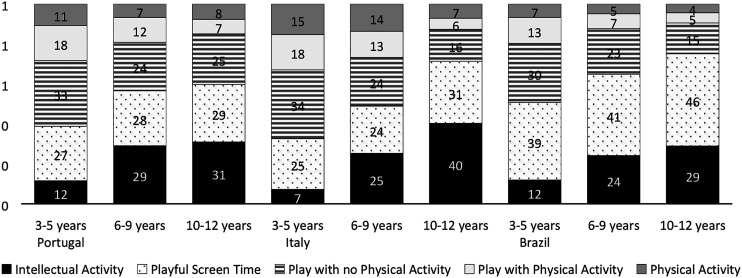
Mean Percentage of Time Children Spent in Different Activities as Reported by Parents.

**Figure 2.**
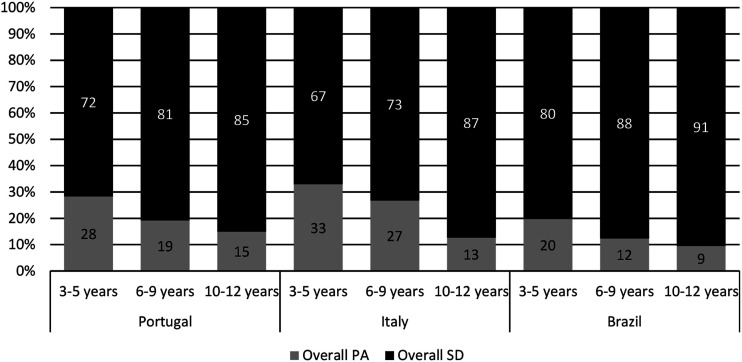
Overall Physical Activity and Sedentary Time.

**Figure 3.**
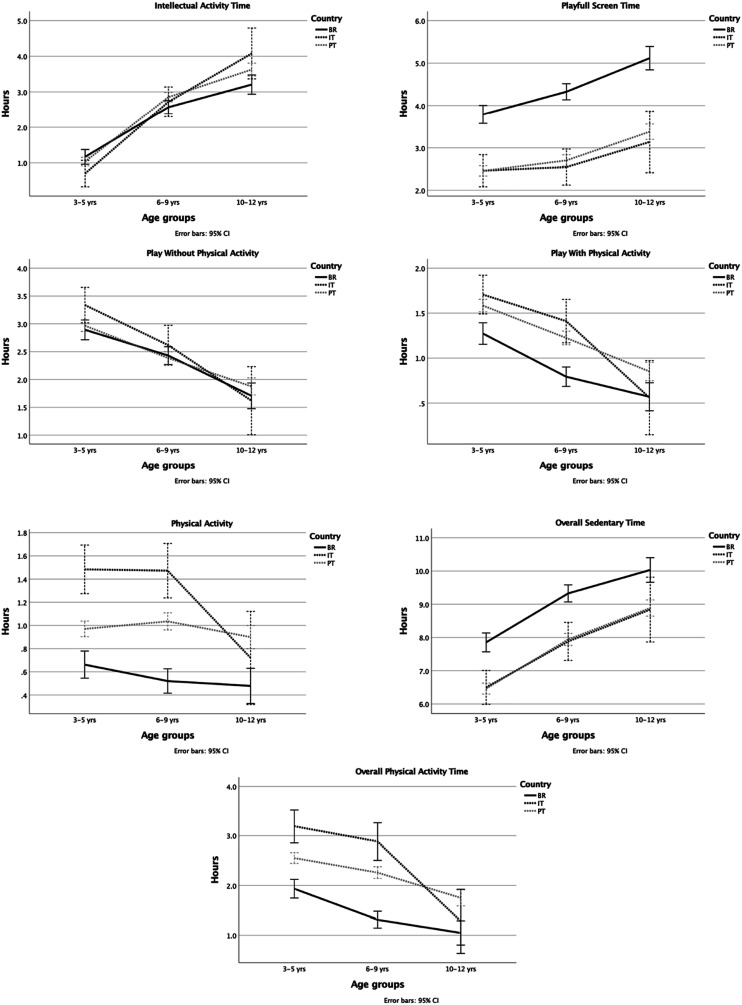
Children’s Average Time (hours) in Different Activities During Social Confinement by Age and Country as Reported by Parents. *Note*. Error bars represent 95% CI.

Overall, our results showed a clear behavior pattern. During lockdown most children spent most of their awake daily hours in sedentary activities. Brazilian children spent less of their time in overall PA than their European peers (Figures 1 and 2). Increased Intellectual Activity, Playful Screen time and Overall Sedentary time was evident across the age groups in all countries, while PA categories showed the opposite trend (i.e., for categories of play without physical activity, play with physical activity, physical activity, and overall physical activity). Interaction effects between countries and age groups were found for Intellectual Activity time, Physical Activity time and Overall Physical Activity time; therefore the behaviors in these categories were not similar across age groups in the different countries (Figure 3 and Table 2). Table 2 provides details regarding the results of ANOVAs.

**Table 2.** ANOVA Results for the Effect of Age and Country and Their Interactions on Groups of Children’s Daytime Activities as Reported by Parents.

Intellectual Activity	Playful Screen Time	Play Without Physical Activity Time	Play With Physical Activity Time	Physical Activity Time	Overall Physical Activity Time	Overall Sedentary Time
Fcountry (2,3047) = 2.999, *p* = .049, η_p_^2^ = 0.120	Fcountry (2, 3047) = 197.764, *p* < .001, η_p_^2^ = 0.115	Fcountry (2, 3047) = .965, *p* = .381, η_p_^2^ = 0.001	Fcountry (2, 3047) = 28.902, *p* < .001, η_p_^2^ = 0.019	Fcountry (2, 3047) = 52.984, *p* < .001, η_p_^2^ = 0.034	Fcountry (2, 3047) = 62.419, *p* = .000, η_p_^2^ = 0.039	Fcountry (2, 3047) = 75.639, *p* < .001, η_p_^2^ = 0.047
Fage (2,3047) = 207.956, *p* < .001, η_p_^2^ = 0.002	Fage (2, 3047) = 19.820, *p* < .001, η_p_^2^ = 0.013	Fage (2, 3047) = 56.001, *p* < .001, η_p_^2^ = 0.036	Fage (2, 3047) = 51.486, *p* < .001, η_p_^2^ = 0.033	Fage (2, 3047) = 8.567, *p* < .001, η_p_^2^ = 0.005	Fage (2, 3047) = 38.494, *p* = .000, η_p_^2^ = 0.025	Fage (2, 3047) = 80.543, *p* < .001, η_p_^2^ = 0.050
Fcountry*age (4,3047) = 3.626, *p* = .006, η_p_^2^ = 0.005	Fcountry*age (4,3047) = 1.413, *p* = .277, η_p_^2^ = 0.002	Fcountry*age (4,3047) = 1.107, *p* = .351, η_p_^2^ = 0.001	Fcountry*age (4,3047) = 1.692, *p* = .149, η_p_^2^ = 0.002	Fcountry*age (4,3047) = 3.318, *p* = .010, η_p_^2^ = 0.004	Fcountry*age (4,3047) = 3.681, *p* = .005, η_p_^2^ = 0.005	Fcountry*age (4,3047) = 3.626, *p* = .914, η_p_^2^ = 0.000

Moreover, we found a main effect of age groups in all categories except that, for PA time, there were no differences between the two younger age groups. Thus, older children presented less time in active behaviors than younger children (i.e. more time in sedentary behaviors and less time in PA). Additionally, for all categories except Play without PA, there was a main effect of country (Table 2).

Regarding Intellectual Activity, the three countries displayed a similar increasing trend for age groups, meaning that there was more time spent in Intellectual Activity for older children. In Italy, this trend was clearer, since Italian children had lower values of Intellectual Activity in the younger age group and higher values in the older age group in comparison with the other countries. However, when comparing countries, only Brazil and Portugal differed significantly from each other in the middle (*p* = .045) and older (*p* = .032) age groups, with Portugal showing higher values of Intellectual Activity. Concerning time spent in Playful Screen activities and Overall Sedentary time, we found similar results. Portuguese and Italian children showed the lowest values, similar across age groups; on the other hand, Brazilian children had significantly higher values than their peers (*p* < .001). In the category, Play with PA, Brazilian children displayed significantly lower PA results than the other countries (*p* < .001), and there were similar values between them. Furthermore, in Physical Activity time and Overall Physical Activity time categories, Portuguese children had significantly higher PA results than Italy and Brazil in all age groups (*p* < .001). Also, Italy presented significantly higher PA values than Brazil in the two younger age groups (*p* < .001).

## Discussion

Our results illustrate that, although the effects of the COVID-19 pandemic on children’s PA were global, they were experienced differently across different countries, seemingly reflecting the variant policies and cultural patterns of each society. The “Little Big Kids” project, carried out by Viacom in 12 countries from four different continents, reported that Brazilian children had an average global screen time that was 50% higher than children from other countries (Viacom, 2017). The pandemic-related social distancing situations led to an even longer screen exposure time for Brazilian children (Mata et al., 2020). In fact, Brazilian children who were more than three years old spent about 2.5 hours per day of screen activity prior to the pandemic (De Araújo et al., 2018), and these numbers increased with the isolation scenario such that higher playful screen time led to an increased sedentary time and a reduced total PA time for these children (Bonilha et al., 2021).

Before the pandemic, Brazilian parents had already shown a lack of will to increase their children’s opportunities to be active. Relevant contributing factors to increased sedentary behavior were concerns about safety preventing the kids from performing outdoor activities, a high demand of activities related to the parents’ jobs, unfavorable structural conditions for PA in specific neighborhoods, and great availability of computer games and TV shows. This context of restriction and the emotional and psychological burdens of guardians or parents, an accumulation of work done from home, parental assumptions of greater responsibilities in their children’s education, and concerns about an uncertain period of the pandemic negatively impacted the children’s support system and care practices (Goncalves et al., 2020; Oliveira, 2020). Also, with all residents staying at home full- time, there was an increase in noise complaints, including noises from children playing and carrying out other activities; this led to Brazilian Federal Law No. 3688 (Brazilian Federal Law No. 3, n.d.) requiring families who lived in apartments to limit the noise of their children, further reducing children’s physical and playful activities within homes in the absence of their access to large external spaces.Although mandatory since kindergarten (4–5 years of age), physical education in Brazil is still seen to be responsible only for the practice of sports training and recreational and/or leisure practice (Barbosa, 2001). It is even perceived as a moment of playing senseless games (Mattos & Neira, 2000) and this cultural devaluing of PA in Brazilian society can help explain our results.

Prior to the COVID-19 lockdown, only 36% of Portuguese children aged 10–11 years accomplished the WHO PA guidelines of 60 minutes per day of moderate-to-vigorous PA (Baptista et al., 2012) and, although 61.8% of 6-9-year-old Portuguese children practice some form of organized sports at least once per week (Lopes et al., 2017), school is still the main place for children’s PA behaviors. In Portugal, Physical Education (PE) classes are mandatory for all students, from pre-school until the 12th grade. Time allocated to PE classes ranges from 90 to 150 minutes/week over 2–3 sessions/week and are taught by a certified PE teacher (Mota et al., 2018).

In Italy, children’s pre-Covid PA levels were estimated to be low by indirect observation (Ministry of Health, 2019). Indeed, while no data are available on time spent in moderate-to-vigorous PA, surveys performed in the last 10 years indicate that more than 40% of 3–9 year old Italian children spent more than two hours/day in sedentary activities and that half of these children had a TV in their sleeping room (INFOGRAFICA RISULTATI OKKIO-DEFINITIVA | Enhanced Reader, 2019). There is no PE in the Italian kindergartens curriculum, and, PE in elementary school is only for two hours/week (and may be reduced by the teachers’ board as needed); it is carried out by generalist teachers. In addition, opportunities for being physically active during leisure time at school are low, due to limited space and to minimal PA-promotions from Italian teachers/Ministry (Tortella et al., 2012). Perhaps to compensate for these PA promotional weaknesses, 65% of the Italian families rely on sport associations to gain PA opportunities for their children (INFOGRAFICA RISULTATI OKKIO-DEFINITIVA | Enhanced Reader, 2019). This minimalist PA attitude seems to be part of a major cultural problem. In Italy; two thirds of the child population commutes to school by motorized means, and parents’ perceptions of their children’s PA levels is over-estimated, even for mostly sedentary and obese children (INFOGRAFICA RISULTATI OKKIO-DEFINITIVA | Enhanced Reader, 2019). Thus, the limited impact of the Covid-19 lockdown on PA levels among Italian children that we observed in this study, may reflect this cultural difference in valuing PA. In Italy, the lockdown began on March 12, 2020, and schools of all levels were closed until September 14, 2020. From March to June (school period) children stayed at home and lessons continued with distance learning that took place every day in middle school and elementary school, within regularly scheduled appointments (15 hours/week). In kindergarten there was no distance learning, and children aged 0–6 years old were free to play in their homes, while older children spent many hours on the computer for daily school appointments. This could explain differences in PA activity in Italy compared to other countries in our studies, and it might also explain both the increase in sedentary behavior in older children compared to younger ones and the lower intellectual activity in the younger age group compared to older children.

Other important variables studied in this sample should be highlighted. A large external space at home for PA activity has been found to influence percentage of children’s PA performed in that children who lived in houses with a big outdoor space (i.e., more than 12 m^2^) were more active (Bonilha et al., 2021; Pombo et al., 2020). Only 17.9% of the Brazilian children in our sample lived in houses with a big outdoor space. This percentage was 36.0% for Portuguese children and 43.3% % for Italian children (Pombo et al., 2020; Sá et al., 2020). Other correlates of PA might also help explain the greater sedentary lifestyle that Brazilian children experienced, compared to children in Portugal and Italy during this period. For example, in Portugal quickly accessed outdoor play time was still allowed during the lockdown, but this access was not permitted in Brazil.

While social isolation was seen as a necessary and effective strategy to prevent the human-to-human transmission of COVID-19, at what cost was this viral safety achieved? Our results showed a clear pattern of sedentary behaviors, which in these ages have been associated with inadequate body composition, decreased fitness, lower self-esteem and pro-social behavior, and decreased academic achievement (Tremblay et al., 2011). Reducing children’s sedentary behavior is an important goal, not only for the prevention and treatment of childhood obesity (Epstein et al., 2000), but also to help to satisfy some of the children’s basic psychological needs, like social connected-ness, self-acceptance, and purpose in life (Rodriguez-Ayllon et al., 2019). In addition increasing the levels of PA, can provide protection from viral infections (Laddu et al., 2020), and it seemed to be associated with a lower prevalence of COVID-19-related hospitalizations (Ribeiro de Souza et al., 2020). For that, governments, health directorates and communities in general should reinforce the message that the use of appropriate outdoor socio-physical environments for play, movement and physical activity is a key measure for children and families to adopt in times of uncertainty and challenge, such as those created by the actual pandemic, so that today’s active children are tomorrow’s healthy adults.

### Limitations and Directions for Further Research

Among this study’s limitations, we should note that we relied on parents’ estimates of their children’s engagement in changes in PA and/or sedentary time during COVID-19 lockdown. Therefore, these data reflect PA estimates that are linked to parents’ perceptions and beliefs about the significance of PA for child development. A stronger belief in the beneficial effects of physical movement on child development would likely have made parents more concerned about the potential negative impact of PA limitations imposed of by lockdown, perhaps affecting their reports of their children’s PA. An online parental report can be less accurate than a direct or quantifiable observation of the time children spent in each activity. Improved methodology in future studies is recommended.

Although this study provides important information considering the differences of the routines of Brazilian, Portuguese and Italian children during lockdown, this was a cross-sectional design study susceptible to some biases. Some methodological characteristics were unavoidable, but the fact that the number of respondents in each country was quite different is also a limitation, meaning that results should be interpreted with caution. For example, we had only 25 participants in the Italian 10–12 year old group, and they may not be representative of this population. In fact, we cannot besure that we had a representative sample in any of the participating countries; the survey does not provide information about the specific place of residency of the participants in each country. Also, due to the high volume of children without internet access (Cetic.br/UNESCO, 2019), Brazilian data cannot be representative of all social classes. Additionally, we could not collect any data on the socio-economic status (SES) of the respondents. We had some indirect indicators, such as the type of house, but we could not specify the respondent’s SES, even though SES is probably an influential factor in the way families dealt with the confinement. Larger participant samples in which these key variables of interest are better controlled are recommended.

## Conclusion

In sum, the results of this study suggest that the effects on children’s PA and sedentary activity during the global COVID-19 pandemic varied across different countries. We found a sample of Brazilian children to have had generally higher overall sedentary activity and generally lower overall PA, than either Portuguese or Italian children, during the first lockdown period of the COVID-19 pandemic. These results are in line with previous PA levels found by earlier studies prior to the lockdown; Brazilian adults, both women and men, had a higher rates of physical inactivity than those of many other countries, including Italy and Portugal (Guthold et al., 2018). The child population of this study during the COVID-19 lockdown seems to reflect the earlier behavior patterns of the adult Brazilian society. Overall, the pandemic had a harmful impact on children’s PA (Moore et al., 2020; Pietrobelli et al., 2020; Pombo, Luz, Rodrigues et al., 2021; Sá et al., 2020), motor competence (Pombo, Luz, de Sá et al., 2021) and even their psychological health (Panda et al., 2021) worldwide. It will be important to follow up these data with future studies to prevent future damage to these affected children.
